# Prevalence and associated factors of diabetic retinopathy among people with diabetes screened using fundus photography at a community diabetic retinopathy screening program in Nepal

**DOI:** 10.1186/s12886-023-03173-z

**Published:** 2023-10-23

**Authors:** Raba Thapa, Sanjita Sharma, Eli Pradhan, Sushma Duwal, Manish Poudel, Krishna Gopal Shrestha, Govinda Prasad Paudyal

**Affiliations:** Tilganga Institute of Ophthalmology, Kathmandu, PO Box: 561, Nepal

**Keywords:** Diabetic retinopathy, Sight threatening diabetic retinopathy, Associated factors, Screening, Nepal

## Abstract

**Background:**

This study aimed to assess the prevalence and associated factors of diabetic retinopathy (DR) and vision threatening DR (VTDR) among people with diabetes screened using fundus photography in Nepal.

**Methods:**

This is a retrospective study among people with diabetes presented for DR screening using fundus photography from 2013 to 2019. Detailed demographics, duration of diabetes, medical history, visual acuity, and grading of DR on fundus photography were analyzed. Fundus camera used in the study were;Topcon digital fundus camera 900 CXR and digital portable fundus cameras (Nidek-10 portable non-mydriatric fundus camera; Versacam & Trade & Alpha, France), and a Zeiss portable fundus camera (Zeiss Visucout 100). Macula centred and disc centred 45 degree two images were taken from each eye. Pupil were dilated in cases where there was media haze in un-dilated cases. DR was graded using early treatment diabetic retinopathy study criteria. The images were graded by fellowship trained retina specialist. DR prevalence included any DR changes in one or both eyes.

**Results:**

Total of 25,196 patients with diabetes were enrolled. Mean age was 54.2 years with Standard Deviation (S.D):12.9 years, ranging from 6 years to 97 years. Type 1 and type 2 diabetes comprised of 451 people (1.79%) and 24,747 (98.21%) respectively. Overall, 1.8% of the images were un-gradable. DR prevalence was 19.3% (95% Confidence Interval (CI): 18.8 − 19.7%). DR prevalence in type 1 and type 2 diabetes was 15.5% (95% CI: 12.5 − 18.6%) and 19.3% (CI: 18.8 − 19.8%) respectively. Clinically significant macular edema (CSME) was found in 5.9% (95% CI: 5.6-6.2%) and VTDR in 7.9% (95% CI: 7.7-8.3%). In multivariate analysis, our study revealed strong evidence to suggest that there is meaningful association between DR and VTDR with duration of diabetes, diabetic foot, diabetic neuropathy, agriculture occupation, those under oral hypoglycaemic agents or insulin or both as compared to those under diet only, and presenting visual acuity > 0.3LogMAR.

**Conclusion:**

Prevalence and associated factors for DR and VTDR were similar to other DR screening programs in the region. Emphasis on wider coverage of DR screening could help for timely detection and treatment of STDR to avoid irreversible blindness.

## Background

Diabetic retinopathy (DR) is an emerging public health problem in low and middle in-come countries. Despite blindness from DR being preventable, it has been the most common cause of blindness among the working aged people in the developed world and the 5th leading cause of global blindness [1]. More than 80% of blindness from DR have been reported from developing countries. People with diabetes are expected to increase by 69% in low middle income countries (LMIC) as compared to only 20% in high income countries from 2010 to 2030 [2]. If not tackled on time, this may pose significant problem in LMIC.

Overall, one-third of people with diabetes have DR, and among those affected by DR, one third have sight-threatening DR (STDR) [3]. Patients with DR are asymptomatic until the advanced stage of STDR. There is limited access to eye care services in the rural areas and trained human resources are urban centered. These factors have caused late presentation of patients with STDR where visual recovery is challenging and can lead to irreversible blindness [4]. Late presentation has also been identified due to lack of public awareness, knowledge and social deprivation [5, 6]. Early detection and timely treatment of STDR could save the vision of people with DR [4]. The study has also demonstrated the relationship between macular perfusion at both retinal and choroidal levels and the cone mosaic in patients with type 1 diabetes. In non-proliferative diabetic retinopathy (NPDR) eyes, the photoreceptor damage was accompanied by choriocapillary circulation insufficiency since the early stages of the disease [7].

The resulting visual impairment and blindness further predispose person to poverty due to loss of productivity, more investment in treatment and poor quality of life and finally become a burden for the nation [8, 9].Regular screening for DR could help in its timely detection. Due to limited work force and increased number of people with diabetes, DR screening using fundus photography, preferably non-mydriatric fundus photography and task shifting is widely practiced globally [8, 10]. These advantages of fundus photography have helped in DR integration in comprehensive diabetes management and also an opportunistic screening of other sight threatening retinal diseases [11–14]. Wider-scale DR screening program is essential to detect STDR for timely treatment. Studies have reported that screening closer to people and/or addition of laser among those with STDR in screening improves compliance, is cost-effective, and helps improve overall quality of life [15, 16].

DR prevalence has been reported to be from 21.7 to 33% among people with diabetes in DR screening program in neighboring Asian countries [17, 18]. International Diabetes Federation (IDF) recently reported an overall prevalence of DR of 27% in DR screening program using fundus photography. The lowest prevalence was in South East Asia (12.5%) and highest was in Pacific Region (36.2%) [19]. Duration of diabetes, poor control of blood sugar, and concurrent hypertension have been reported as the three major risk factors for DR. Likewise, other factors like hyperlipidaemia, type of diabetes, smoking, anemia, pregnancy and nephropathy have also been identified as important risk factors for early onset and progression of DR [3, 20–23]. Previous studies of prevalence and risk factors of DR and VTDR in Nepal were conducted in small sample size, mostly from local areas [23–27]. There are limited regular community DR screening programs in Nepal and DR screening services are provided mainly from tertiary eye hospitals.

This study aimed to assess the prevalence of DR and associated factors of DR among large number of people with diabetes who participated in DR screening program using both mydriatric and non-mydriatric fundus photography in Nepal.

## Materials and methods

This is a retrospective case-series study conducted among people with diabetes at diabetic retinopathy screening program (DRP) under Tilganga Institute of Ophthalmology (TIO) in collaboration with other organizations. TIO, a tertiary eye care center, is a non- governmental organization in Nepal. It has its own primary and secondary eye care centers in various parts of the country. People have to pay for DR screening and treatment at tertiary and secondary eye care centers. As a part of DR screening program, in primary eye care centers and other screening sites, DR screening and treatment was conducted for free with the support of the funding agencies.

All the consecutive cases screened at DRP with collaborating public hospitals, secondary community eye hospital, primary eye care centers, professional diabetes society, diabetes association and other diabetic retinopathy screening camps were included in the study. This study was approved by Tilganga Institute of Ophthalmology- Institutional Review Committee (TIO- IRC) (11/2020), on 28 May 2020 Kathmandu, Nepal. The need for written informed consent was waived by the TIO-IRC ethics committee due to retrospective nature of the study. Study was conducted in accordance to Declaration of Helsinki.

A total of 25,228 people with diabetes were screened in the DR screening program. Major parts of information were missing in 30 cases. The missing data were for detail demographic information, vision and fundus pictures. After excluding these missing data, a total of 25,196 people with diabetes were enrolled in the study. Detailed information was collected from the DR screening record paper of each patient. The information comprised of demographic characteristics, type of diabetes, duration of diabetes, concurrent hypertension, hyperlipidaemia, cardiac diseases, anaemia, renal problems, neuropathy, pregnancy, details of treatment like diet only, oral hypoglycaemic agents (OHA), insulin, etc. Examination findings recorded were visual acuity, grading of DR, and information of non-gradable fundus photographs. Visual acuity was taken at the screening sites. Uncorrected visual acuity, presenting visual acuity with glass and best corrected visual acuity using pin hole were assessed using Snellen chart and or standard visual acuity chart available at the screening sites.

The fundus pictures were captured using both desktop (Topcon digital fundus camera 900 CXR) and digital portable fundus cameras. The two types of digital portable fundus cameras used were Nidek-10 portable non-mydriatric fundus camera (Versacam& Trade & Alpha, France), and a Zeiss portable fundus camera (Zeiss Visucout 100). The non mydriatric fundus pictures were taken. In the cases of people with diabetes having media haziness, unable to fix the eyes, and more artifacts, mydriatric fundus pictures were taken.

Inclusion criteria: People with diabetes screened using either mydriatric or nonmydriatric fundus camera under DRP from 2013 to 2019. Exclusion criteria: People with diabetes screened for DR but missing significant parts of demographic information and fundus pictures.

### Training of fundus photographers

Fundus pictures were taken by the trained allied ophthalmic personnel (AOP), allied medical personnel (AMP) and ophthalmic photographers [10, 12]. In brief, AMP had received either government-certified degrees in nursing, and or certificates in general medicine. The overall duration of training period was six-months, which included both theoretical and practical sessions. The theoretical training was of 3 weeks and mainly focused on teachings of overall anatomy, physiology of the eye, ocular and emphasis on retinal pathologies. The training period was scheduled as seven hours during working days for 6 months. AOP had completed government certified course to provide primary eye care in ophthalmology and were trained on DR grading. They received one week of theoretical and six months of practical session on fundus photography.

### Fundus photography

Fundus pictures were taken using either Topcon digital fundus camera or portable fundus cameras by the trained AOPs and trained AMPs. In non mydriatric cases, one macula centered and one optic disc centered fundus pictures were taken from all eyes and enrolled in the study. Fundus was dilated where media was hazy, patients were un-cooperative and when fundus photographs were not clear enough for grading in non-mydriatric fundus photography. Five fundus pictures were taken under mydriasis that included one macula centered, one optic disc centered, one supero-temporal, one infero-temporal and one nasal to optic disc. Either mydriatric or non-mydriatric fundus cameras were used in DR screening program at various locations as per the availability. All the people with diabetes screened using fundus camera irrespective of the type of fundus camera and pupil status were enrolled in the study.

In this DRP, 1.8% of the images of one or both eyes were un-gradable. DR was diagnosed if any DR changes were present in either one eye or both eyes.

For DR screening, two fields were used while using non-mydriatric fundus camera and five fields were used in mydriatric eyes. Mydriatric fundus photographies were taken only when non-mydriatric fundus photographs were ungradable due to media haze, artifacts or difficulty in focus by the patients.

### Fundus photo grading

Fundus photographs were graded by fellowship trained retina specialist. DR was graded using Early Treatment Diabetic Retinopathy Study (ETDRS) criteria in fundus photographs [4]. In brief, DR was graded as non-proliferative diabetic retinopathy (NPDR) and proliferative diabetic retinopathy (PDR). NPDR was further classified as mild NPDR, moderate NPDR, severe NPDR and very severe NPDR. Diagnosis of clinically significant macular edema (CSME) in fundus photographs was based on presence of hard exudates at or within 500 μm of the center of macula, or a presence of hard exudates larger than one disc area if located within one disc diameter of the center of the macula. The presence of exudates was taken as a criteria for defining CSME in fundus photography. Separate red free images were not available for the graders to assess CSME.

VTDR was considered for those with any stage of severe NPDR, very severe NPDR, PDR and or CSME in at least in one eye. When detailed findings of DR were not discernable on fundus photographs, the results were mentioned as un-gradable. If the fundus photographs were clear enough for grading the signs of DR, then they were taken as gradable fundus photographs. While calculating DR prevalence, CSME was not included in the DR classification.

While calculating DR prevalence in a person, the eye with severe retinopathy was considered. For example, if one eye had severe NPDR and fellow eye had moderate NPDR, DR was considered severe NPDR in the person for DR prevalence calculation.

### Statistical analysis

Descriptive statistics such as frequencies, percentages (with 95% confidence interval), mean; standard deviation: (SD) etc. were determined. For categorical data analysis, Chi Square or Fisher’s Exact test were used wherever applicable. For numerical, normally distributed data, student test were used. Univariate and multiple logistic regression analysis were done to identify significant associated factors for DR and VTDR separately. All the statistically significant independent variables in the univariate analysis were considered as candidate variables for multiple logistic regression analysis. P value was considered statistically significant if it was less than 0.05. Statistical Package for Social Sciences (SPSS) V.19 was used for the data analysis. Data was collected from 16 different hospitals/clinic sites/primary eye care centers. These DR screening sites were coded by numbers ranging from 1 to 16. Participants were given code numbers from 1. In excel file, separate sheets were used for coding the patients and hospitals. The data and code are kept in software protected computer. Patients’ confidentiality was maintained.

## Results

Among the total collected data 25,228; 30 were missing. Some data did not have demographic information, vision and some have only demographic information like gender. After excluding these missing data, 25,198 people with diabetes were taken for the analysis in this study. Type 1 and type 2 diabetes comprised of 451 people (1.79%) and 24,747 (98.21%) respectively. The overall mean age was 54.2 years (S.D:12.9years) ranging from 6 years to 97 years. The average age (SD) of type 1 DM and type 2 DM were 25.8 (9.4) years and 55.2 (11.8) years respectively.

Majority of the people with diabetes were from the age group 51 to 55 years (14.8%), followed by 46 to 50 years and 56 to 60 years comprising of (14.5%) each. Males were more in number (51.3%). Housewives were the majority (47%), followed by service holders (12.6%), businesses (11%) and agriculture (9.6%) who presented for DR screening. Similarly, Janajati (43.4%) and hill caste (43.3%) were the major ethnic groups presented for DR screening. Among the 25,198 people with diabetes screened for DR, gradable fundus photographs were found in 24,750 (98.2%) and fundus photographs were non-gradable in 448 (1.8%). Those people with non-gradable fundus photographs were significantly older (p < 0.001) as compared to gradable fundus photographs. (Table 1)

**Table 1:** Age and gender distribution of people with diabetes having gradable and non-gradable fundus photographs

Variable	Gradable fundus photos (n = 24,750)	Non-gradable fundus photos (n = 448)	p value
Age, mean †(SD) in years	54.1 (12.9)	57.5 (14.5)	< 0.001
Male, ‡n (%)	12,710 (51.4)	224 (50)	0.57
Female, ‡n (%)	12,040 (48.6)	224 (50)	

†Standard Deviation; ‡ Number

The overall prevalence of DR was 19.3% (95% Confidence Interval (CI): 18.8 − 19.7%). It was found slightly higher among males (20.5%, 95% CI: 19.8 − 21.2%) as compared to females (17.9%, 95% CI: 17.3 − 18.6%), and those of 50 years and above age group (21.5%, 95% CI: 20.9 − 22.2%) as compared to those below 50 years of age (14.9%, 95% CI: 14.2 − 15.7%).

DR was found in 9.4% (95% CI: 7.6 − 11.3%) among those who were of ages 30 years old and below while it was found in 21.5% (95% CI: 20.6 − 22.5%) among those over 60 years of age. DR was found in 13.9% (95% CI: 13.4 − 14.4%) among those with diabetes duration of less than 10 years. DR was found in 47.9% (95% CI: 43.9 − 51.8%) among those with more than 20 years of diabetes duration. The prevalence of DR in type 1 and type 2 diabetes was 15.5% (95% CI: 12.5 − 18.6%) and 19.3% (95% CI: 18.8 − 19.8%) respectively. DR in diet control only group and treatment receiving group was 7.8% (95% CI: 7 − 8.6%) and 21.5% (95% CI: 21 − 22.1%) respectively. Among treatment receiving group, DR in oral hypoglycaemic agents (OHA), insulin and both (OHA with insulin) were 19.9% (95% CI: 19.3 − 20.4%), 26.3% (95% CI: 23.6 − 29.1%) and 44.1% (95% CI; 41.3 − 47%) respectively. (Table 2)

**Table 2:** Distribution of prevalence of diabetic retinopathy among various groups

Variables	No DR^†^ n^‡^(%)	Mild NPDR^§^ n (%)	Moderate NPDR n (%)	Severe + very severe NPDR n (%)	PDR^¶^ n (%)	DR (n = 4766) % (95% CI)
**Total**	19,984 (80.7)	1707 (6.9)	1933 (7.8)	717 (2.9)	409 (1.7)	19.3 (18.8–19.7)
**Gender**						
Male	10,104 (79.5 )	891 (7 )	1071 (8.4 )	409 (3.2)	235 (1.8 )	20.5 (19.8–21.2)
Female	9880 (82.1 )	816 (6.8 )	862 (7.2 )	308 (2.6)	174 (1.4 )	17.9 (17.3–18.6)
**Age group**						
< 50	7232 (85.1 )	546 (6.4 )	477 (5.6 )	169 (2.0)	77 (0.9 )	14.9 (14.2–15.7)
50 and above	12,752 (78.5 )	1161 (7.1 )	1456 (9 )	548 (3.4)	332 (2 )	21.5 (20.9–22.2)
Up to 30	853 (90.6)	42 (4.5)	32 (3.4)	7 (0.7)	8 (0.8)	9.4 (7.6–11.3)
31–60	13,124 (81.3)	1134 (7)	1166 (7.2)	469 (2.9)	258 (1.6)	18.7 (18.1–19.3)
> 60	6007 (78.5)	531 (6.9)	735 (9.6)	241 (3.1)	143 (1.9)	21.5 (20.6–22.5)
**DM duration (years)**						
< 10	16,510 (86.1)	1149 (6)	1015 (5.3)	345 (1.8)	156 (0.8)	13.9 (13.4–14.4)
11–20	3155 (63.6)	503 (10.1)	783 (15.8)	326 (6.6)	196 (3.9)	36.4 (35.1–37.8)
> 20	319 (52.1)	55 (9)	135 (22.1)	46 (7.5)	57 (9.3)	47.9 (43.9–51.8)
**DM type**						
Type 1	451 (84.5)	33 (6.2)	33 (6.2)	8 (1.5)	9 (1.7)	15.5 (12.5–18.6)
Type 2	19,533 (80.7)	1674 (6.9)	1900 (7.8)	709 (2.9)	400 (1.7)	19.3 (18.8–19.8)
**Treatment**						
Yes	16,192 (78.5)	1542 (7.5)	1821 (8.8)	685 (3.3)	397 (1.9)	21.5 (21–22.1)
No (Diet only)	3792 (92.2)	165 (4.0)	112 (2.7)	32 (0.8)	12 (0.3)	7.8 (7–8.6)
**Types of treatment**						
Diet	3792 (92.2)	165 (4.0)	112 (2.7)	32 (0.8)	12 (0.3)	7.8 (7–8.6)
OHA	14,812 (80.1)	1366 (7.4)	1489 (8.1)	539 (2.9)	278 (1.5)	19.9 (19.3–20.4)
Insulin	733 (73.7)	73 (7.3)	104 (10.5)	52 (5.2)	33 (3.3)	26.3 (23.6–29.1)
OHA with Insulin	647 (55.9)	103 (8.9)	228 (19.7)	94 (8.1)	86 (7.4)	44.1 (41.3–47)

Note: 448 (1.8%) were non-gradable

†*diabetic retinopathy;* ‡ *non-proliferative diabetic retinopathy;* § number; ¶ *proliferative diabetic retinopathy*

CSME was found in 5.9% (95% CI: 5.6-6.2%) and VTDR was found in 7.9% (95% CI: 7.7-8.3%) of people who screened for DR. VTDR and CSME were significantly higher among males, 8.7% (95% CI; 8.2-9.2%), and 6.4% (95% CI: 6-6.8%), with p value (< 0.001) respectively as compared to females, 7.3% (95% CI: 6.8-7.7%) and 5.4% (95% CI: 5-5.8%), with p value (p = 0.001) respectively. (Table 3)

**Table 3:** Distribution of VTDR and CSME among people with diabetes screened for DR

Variables	Total		Male		Female		p value
	Count	% (95% CI^§^)	Count	% (95% CI)	Count	% (95% CI)	
VTDR^†^ (Yes)	1977	7.9 (7.7–8.3)	1103	8.7 (8.2–9.2)	874	7.3 (6.8–7.7)	< 0.001
CSME^‡^ (Yes)	1463	5.9 (5.6–6.2)	815	6.4 (6–6.8)	648	5.4 (5–5.8)	0.001

†*Vision threatening diabetic retinopathy;* ‡ *clinically significant macular edema*, § Confidence interval,

In univariate analysis, factors including, male gender (OR, 1.2 compared with female), hypertension (OR, 1.2 compared to non-hypertensive), hyperlipidemia (OR, 1.2 compared to no hyperlipidemia), diabetic neuropathy (OR, 1.6 compared with no diabetic neuropathy), diabetic foot (OR, 1.9 compared with no diabetic foot), age (odds ratio [OR], 1.2 per 10-year increase), agriculture occupation (OR, 1.2 compared with other occupation), duration of diabetes (OR, 1.7 per 5-year increase), presenting visual acuity (PVA) > 0.3 LogMAR(OR, 1.7, compared with PVA < = 0.3LogMAR), OHA (OR, 2.9, compared with diet only), insulin treatment (OR, 4.2 compared with diet only) and both OHA and insulin (OR, 9.3 compared with diet only) were found to be highly associated with odds of developing any type of DR. (Table 4)

Similarly, factors including age (OR, 1.2 per 10-year increase), male gender (OR, 1.2 compared with female), hypertension (OR, 1.3 compared to non-hypertensive), diabetic neuropathy (OR, 1.7 compared with no diabetic neuropathy), diabetic foot (OR, 2.5 compared with no diabetic foot), agriculture occupation (OR, 1.2 compared with other occupation), duration of diabetes (OR, 1.7 per 5-year increase), PVA > 0.3 (OR, 2.6, compared with PVA < = 0.3), OHA (OR, 4.5, compared with diet only), insulin treatment (OR, 7.5 compared with diet only) and both OHA and insulin (OR, 17 compared with diet only) were also associated with vision-threatening DR. (Table 4)

**Table 4:** Associated factors for DR and VTDR among the people with diabetes screened using fundus photographs in univariate analysis

Variables	DR ^†^		VTDR^‡^	
	OR^§^ (95% CI)	P value	OR (95% CI)	P value
Male	1.2 (1.1–1.3)	< 0.001	1.2 (1.1–1.3)	< 0.001
Literate compared with Illiterate	0.9 (0.9–1)	0.201	1 (1–1)	0.806
HTN (yes)	1.2 (1.2–1.3)	< 0.001	1.3 (1.2–1.4)	< 0.001
Hyperlipidemia (yes)	1.2 (1–1.3)	0.036	1.1 (0.9–1.4)	0.289
Heart disease (yes)	1.1 (1–1.3)	0.152	1 (0.8–1.3)	0.775
Diabetic neuropathy (yes)	1.6 (1.4–1.9)	< 0.001	1.7 (1.3–2.1)	< 0.001
Diabetic foot (yes)	1.9 (1.5–2.4)	< 0.001	2.5 (1.8–3.3)	< 0.001
Pregnancy (yes)	0.3 (0–2)	0.218		
Anemia (yes)	1.8 (0.4–7)	0.425	3.2 (0.7–15.4)	0.166
Regular yearly eye examination (yes)	1 (0.9–1.1)	0.420	1.1 (0.9–1.3)	0.229
Age (10 years)	1.2 (1.1–1.2)	< 0.001	1.2 (1.1–1.2)	< 0.001
Age ≥ 50 compared with < 50 years	1.6 (1.5–1.7)	< 0.001	1.7 (1.5–1.9)	< 0.001
Agriculture compared with other	1.2 (1.1–1.3)	0.002	1.2 (1–1.4)	0.012
Diabetes duration (5 years)	1.7 (1.7–1.8)	< 0.001	1.7 (1.7–1.8)	< 0.001
PVA ¶ (> 0.3 compared with ≤ 0.3)	1.7 (1.6–1.8)	< 0.001	2.6 (2.4–2.9)	< 0.001
OHA compared with diet	2.9 (2.6–3.3)	< 0.001	4.5 (3.5–5.6)	< 0.001
Insulin compared with diet	4.2 (3.5–5.1)	< 0.001	7.5 (5.6–10)	< 0.001
Both (OHA and insulin) compared with diet	9.3 (7.9–11)	< 0.001	17 (13.1–22)	< 0.001

†*diabetic retinopathy*, ‡ *vision threatening diabetic retinopathy*, §*Odds ratio*, ¶ *presenting visual acuity*

In multivariable logistic regression analysis, agriculture occupation (OR, 1.2; 95% CI: 1.0–1.4; p = 0.026), diabetic neuropathy (OR, 1.4; 95% CI: 1.1–1.6; p = 0.001), diabetic foot (OR, 1.6; 95% CI: 1.2–2.0; p = 0.001), PVA > 0.3 (OR, 1.2; 95% CI: 1.1–1.4; p = 0.001), duration of diabetes (OR, 1.7; 95% CI: 1.6–1.8; p < 0.001), OHA compared with diet (OR, 2.5; 95% CI: 2.1–3.0; p < 0.001), Insulin compared with diet (OR, 3.4; 95% CI: 2.6–4.5; p < 0.001) and both (OHA and insulin) compared with diet (OR, 5.0; 95% CI: 3.8–6.6; p < 0.001) were significantly associated with DR. (Table 5)

Similarly, agriculture occupation (OR, 1.3; 95% CI: 1.0–1.6; p = 0.033), diabetic foot (OR, 2.0; 95% CI: 1.4–2.8; p < 0.001), PVA > 0.3 (OR, 2; 95% CI: 1.7–2.3; p < 0.001), duration of diabetes (OR, 1.7; 95% CI: 1.6–1.8; p < 0.001), OHA compared with diet (OR, 4.9; 95% CI: 3.2–7.5; p < 0.001), insulin compared with diet (OR, 6.0; 95% CI: 3.6–10.1; p < 0.001) and both (OHA and insulin) compared with diet (OR, 10.9; 95% CI: 6.7–17.8; p < 0.001) were significantly associated with VTDR. (Table 5)

**Table 5:** Associated factors for DR and VTDR among people with diabetes screened using fundus photographs in multivariate analysis

Variables	DR		VTDR	
	OR^†^ (95% CI)	P value	OR (95% CI)	P value
Male compared with female	1.1 (1–1.2)	0.090	1.1 (1–1.3)	0.128
Agriculture compared with Other occupations	1.2 (1–1.4)	0.026	1.3 (1–1.6)	0.033
HTN^‡^ (Yes)	1 (0.9–1.1)	0.581	1 (0.8–1.1)	0.773
Diabetic neuropathy (Yes)	1.4 (1.1–1.6)	0.001	1.2 (1–1.6)	0.096
Diabetic foot	1.6 (1.2–2)	0.001	2 (1.4–2.8)	< 0.001
PVA^§^ (> 0.3 compared with < = 0.3)	1.2 (1.1–1.4)	0.001	2 (1.7–2.3)	< 0.001
Diabetes duration (5 years)	1.7 (1.6–1.8)	< 0.001	1.7 (1.6–1.8)	< 0.001
Age 50 years	1 (0.9–1.1)	0.760	1 (0.8–1.2)	0.774
OHA^¶^ compared with diet	2.5 (2.1–3)	< 0.001	4.9 (3.2–7.5)	< 0.001
Insulin compared with diet	3.4 (2.6–4.5)	< 0.001	6 (3.6–10.1)	< 0.001
Both (OHA and insulin) compared with diet	5 (3.8–6.6)	< 0.001	10.9 (6.7–17.8)	< 0.001
Hyperlipidemia (Yes)	0.9 (0.8–1.1)	0.453		

†*Odds Ratio;* ‡*hypertension*, §*presenting visual acuity*, ¶*Oral hypoglycaemic agents*

## Discussion

This study reported DR prevalence and associated factors involving a large number of people with diabetes screened using fundus photographs in Nepal. In this study, people with type 1 and type 2 diabetes comprised of 1.79% and 98.21% respectively. The mean age of people with diabetes was 54.2 years in our study, which was similar to other population based studies conducted on DR in Nepal [23–25]. Males were slightly higher (51.3%) in our case series unlike in other studies where there was slight female predominance [23–25]. Housewives comprised the highest numbers (45%) unlike in previous population based study where majority were farmers [23].

Non-gradable fundus photographs were found in 1.8% of people screened for DR in our series. The high proportion of gradable fundus photographs in our study could be due to dilatation of pupil for all cases with hazy media and or un-gradable fundus photographs in undilated pupil. In our study, prevalence of any DR was found in 19.3% of people with diabetes screened for DR using fundus photography. The previous study on DR prevalence from Nepal in fundus photograph grading by Mishra et al. [24] which was conducted in urban part of Nepal reported prevalence of DR of 9.9%. The prevalence of DR using fundus photography was higher in our case series. The difference could be due to variation in duration of diabetes and other concurrent risk factors. Our prevalence of DR was also slightly higher as compared to the study conducted in DR screening at eastern part of Nepal (15.3%) on clinical examination [26]. However the prevalence of DR was similar to the population based study by Paudyal et al. [25] (19.3%) and hospital based study by Shrestha et al. [27] (21%) on clinical examination. The similarities in DR prevalence with these two studies could be due to similar geographic location as these two studies were conducted in the capital city and most of our DR screening participants were also from there. International Diabetes Federation (IDF) reported diabetic patients data screened using fundus photography from 2015 to 2019 comprising of 5,43,884 patients. The overall prevalence of any DR among all screened cases for DR was 27%. Among those screened cases, the lowest prevalence was found in South East Asia (12.5%) and highest prevalence of DR was found in Pacific Region (36.2%) [19]. Our prevalence of DR was higher than reported from the South East Asia in this report. The difference could be related to variation in overall age of the patients and duration of diabetes. Gudkari et al. reported DR prevalence in large case series of diabetic patients enrolled for DR screening program in India. A total of 6,218 patients from 194 centers were screened for DR, out of which 5,130 diabetic patients who have adequate information were selected for analysis. The overall prevalence of DR was 21.7% [17]. Our prevalence of DR was consistent with their case series screened for DR. A DR screening program in Bangladesh using fundus photographs that enrolled 49,264 diabetic patients during 7 year period [18]. The overall prevalence of DR was 33%, DR prevalence varied with the location ranged from 13 to 64%. The overall DR prevalence was lower than this series however it was similar to some of the geographical locations. The major difference could be due to variation of concurrent risk factors in these different geographical regions. Our prevalence of DR was slightly higher than one of the population based study from India, a neighboring country [28]. Our prevalence of DR was lower compared to the global DR prevalence of 35.6% and from developed countries [3, 29–32].

VTDR was found in 7.8% of patients in our study. Our findings of VTDR was lower than the VTDR of 9.5% reported in one of the population based study in Nepal among elderly people at the age 60 years and above [23]. The difference could be due to difference in duration of diabetes and lower age in our study with mean age of 55 years as compared to 69 years among elderly age group. Our prevalence of VTDR was also lower than that reported from global VTDR prevalence of 11.72% [3]. However, our prevalence of VTDR was similar to that reported from many countries [33, 34]. Similarly, macular edema was found in 5.8% of patients in our DR screening program. The prevalence of macular edema in our study was higher than that reported from population based study in Nepal with the prevalence of 4.2% in the clinical examination [23]. Over-estimation of macular edema in reading of fundus photographs and or due to difference in other risk factors could be the possibility for this variation. Our prevalence of macular edema was similar to some studies [35, 36].

In our case series, in multivariate analysis, duration of diabetes, diabetic foot, diabetic neuropathy, agriculture occupation, those under OHA or insulin or both as compared to those under diet only, PVA > 0.3LogMAR, were significantly associated with any type of DR and VTDR. Duration of diabetes was a significant associated factor for DR and VTDR in our study, which was consistent with previous studies [3, 20–23]. Presence of diabetic foot was significantly associated with both DR and VTDR. Other studies have reported similar results like in our study [37, 38]. Developing a screening protocol for DR among all cases with diabetic foot could help in timely detection of VTDR and prompt treatment. The recent study has revealed the serum micro-RNA (miRNA) levels could serve as a potential novel biomarker for the early detection of DR in individuals with diabetes. These miRNA levels could also serve as non-invasive targets for therapeutic interventions aimed at halting the progression of the disease during its initial stages [39]. Diabetic neuropathy is one of the most commonly seen complications of diabetes. Our study revealed strong evidence to suggest that there is a meaningful association between DR and diabetic neuropathy. These findings were consistent with other studies [40]. Those who are under regular medications had DR as compared to those under diet only. In our series, people under diet only were those with high blood sugar on and off. So, those under the treatment group having significantly higher DR and VTDR could be related to the duration of diabetes and also poor glycaemic control over years. Those who have agricultural background had significantly more DR and VTDR. This could be related to poor control of blood sugar and or inappropriate treatment of diabetes. The severity of DR and VTDR are mostly associated with visual impairment. So, this fact explains how those who have poor visual acuity had significantly more DR and VTDR.

Quality of retinal images is very important for accurate grading of the DR. In our study, 1.8% of the fundus photographs were un-gradable. As compared to other studies, our fundus photographs were more gradable [41, 42]. The lower rates of un-gradable fundus photographs may have been due to mydriatric fundus photographs wherever there was hazy media.

Strength of the study includes enrolment of large number of people with diabetes including detailed information for DR screening using fundus photography. The findings could be a useful guide for further DR intervention programs for reducing blindness from DR Nepal and other similar countries in the future.

## Conclusion

DR was found in four fifths of patients with diabetes who presented for DR screening at a community DR screening program in Nepal. Our prevalence and associated factors for DR were similar to other studies and similar to DR screening program from many other countries. Emphasis on wider coverage of DR screening could help for timely detection and treatment of STDR to reduce the blindness from DR.

## Data Availability

All data related to study have been included in the manuscript. The principal investigator (RT) will provide further information as needed.

## Abbreviations

DR: Diabetic Retinopathy
LMIC: Low middle income countries
STDR: Sight threatening diabetic retinopathy
TIO: Tilganga Institute of Ophthalmology
DRP: Diabetic retinopathy screening program
IRC: Institutional review committee
OHA: Oral hypoglycaemic agents
AOP: Allied ophthalmic personnel
AMP: Allied medical personnel
ETDRS: Early Treatment Diabetic Retinopathy Study
PDR: Proliferative diabetic retinopathy
NPDR: Non-proliferative diabetic retinopathy
CSME: Clinically significant macular edema
SD: Standard deviation
CI: Confidence interval
DM: Diabetes mellitus
IDF: International Diabetes Federation
